# Supplementary material for: The Complete Chloroplast Genome of Two Important Annual Clover Species, Trifolium alexandrinum and T. resupinatum: Genome Structure, Comparative Analyses and Phylogenetic Relationships with Relatives in Leguminosae
Source: Plants (Basel). 2020 Apr 9;9(4):478. doi: 10.3390/plants9040478 (PMC7238141; doi:10.3390/plants9040478)
Supplement: Supplementary file 1 [file plants-09-00478-s001.zip › Table S1.docx]

Table S1. Location and length of intron-containing genes in the chloroplast genomes of *T. alexandrinum* and *T. resupinatum*

| Gene | *Trifolium alexandrinum* | | | | | *Trifolium resupinatum* | | |
| --- | --- | --- | --- | --- | --- | --- | --- | --- |
|  | Exon Ⅰ (bp) | Intron Ⅰ (bp) | Exon Ⅱ (bp) | Intron Ⅱ (bp) | Exon Ⅲ (bp) | Exon Ⅰ (bp) | Intron Ⅰ (bp) | Exon Ⅱ (bp) |
| *atpF* | 203 | 623 | 412 |  |  |  |  |  |
| *clpP* | 365 | 729 | 220 |  |  |  |  |  |
| *ndhA* | 562 | 1253 | 536 |  |  |  |  |  |
| *ndhB* | 723 | 687 | 756 |  |  |  |  |  |
| *rpoC1* | 9 | 770 | 1629 |  |  |  |  |  |
| *rps18* | 304 | 31 | 14 |  |  |  |  |  |
| *tRNA-CGU* | 36 | 725 | 44 |  |  |  |  |  |
| *tRNA-UAA* | 35 | 608 | 50 |  |  | 35 | 536 | 50 |
| *tRNA-UAC* | 39 | 579 | 35 |  |  | 40 | 577 | 36 |
| *tRNA-UGC* | 37 | 796 | 54 |  |  | 37 | 793 | 54 |
| *tRNA-UUC* | 32 | 681 | 40 |  |  | 32 | 677 | 40 |
| *tRNA-UUU* | 38 | 2459 | 36 |  |  | 37 | 2460 | 35 |
| *ycf3* | 126 | 719 | 228 | 762 | 153 |  |  |  |
