# Supplementary material for: The Complete Chloroplast Genome of Two Important Annual Clover Species, Trifolium alexandrinum and T. resupinatum: Genome Structure, Comparative Analyses and Phylogenetic Relationships with Relatives in Leguminosae
Source: Plants (Basel). 2020 Apr 9;9(4):478. doi: 10.3390/plants9040478 (PMC7238141; doi:10.3390/plants9040478)
Supplement: Supplementary file 1 [file plants-09-00478-s001.zip › Table S2.docx]

Table S2 The shared repeats of *T. alexandrinum* and *T. resupinatum.*

| Units | Times | Number | Location regions (genes) |
| --- | --- | --- | --- |
| A | 8 | 56/57 | *clpP*, *tRNA-UUU*, *rpl23*^*^, *rps3*, *rpoA*, *ycf3*, *tRNA-UGC*, *rrn16*, *ycf1*^*^, *rpoB*, *rpoC2*, *atpF*, *tRNA-CGU*, *psbK*^res^, *ycf2*^res^, *ycf4*^res^, *tRNA-UGC*^res^, *ndhD*^res^, *rpl32*^res^ |
| A | 9 | 32/26 | *clpP*, *matK*, *tRNA-UAC*, *rpoC1*, *tRNA-UAA*, *ndhD*, *rpl32*, *ndhA*, *rpoC2*^res^, *petA*^res^, *ycf1*^res^ |
| A | 10 | 12/26 | *tRNA-UUU, rps19, rpoC2, rps18^res,^ ycf1^res^* |
| A | 11 | 18/22 | *ycf1^*^, rpoC2, ndhl, ndhF^*^, rps15^*^, tRNA-GGU^res^, matK^res^* |
| A | 12 | 7/9 | *ycf3, ycf1^*^, rpoC1* |
| A | 13 | 6/1 | *rpoC2, rps18^res^* |
| T | 8 | 40/57 | *ycf4, ycf1^*^, rpoC1, rpoC2^*^*, *atpF, ndhF^*^, rpoB^res^, rpa3^res^, matK^res^* |
| T | 9 | 23/29 | *rps18, petA, ycf2^*^, tRNA-UAC^*^*, *matK^res^*, *rps7^res^, ycf1^res^* |
| T | 10 | 14/22 | *clpP*, *tRNA-GGU*, *ycf3*, *rpoC2^res^*, *ycf2^res^, rps19^res^, tRNA-UUU^res^, ndhG^res^* |
| T | 11 | 13/11 | *matK*, *rpl32^*^*, *rps18* |
| AT | 5 | 19/7 | *ycf3*, *rpoC2*, *ccsA*, *tRNA-UAA*, *tRNA-CGU*, *rpoB^res^* |
| AAG | 3 | 4/3 | *rps15*, *ycf1*, *tRNA-UAC^res^* |
| AAT | 3 | 8/10 | *ndhA*, *rpl20*, *ndhF^res^*, *tRNA-CGU*, *rpoC2^res^* |
| ACC | 3 | 1/1 | *ycf2^*^* |
| AGA | 3 | 6/5 | *rpoA*, *ndhB*, *ycf1*, *ndhA*, *ccsA^*^* |
| CAA | 3 | 1/2 | *psbB^*^*, *rpl14^res^* |
| CTT | 3 | 1/1 | *tRNA-UAC*, *ycf2^res^* |
| GAA | 3 | 6/4 | *ycf2*, *rpl2*, *ycf1^*^*, *rpoC1* |
| TAA | 3 | 5/6 | *rpoC2*, *petA^res^* |
| TTA | 3 | 5/6 | *ndhF*, *rpoC2^res^*, *rpl14^res^* |
| TTC | 3 | 4/9 | *ycf2*, *ycf1*, *ycf2^res^*, *rpoA^res^* |
| AGGT | 3 | 1/1 | *rrn16^*^* |
| TATT | 3 | 11 | *ndhD^*^* |
| AAGAAC | 3 | 1/1 | *ycf1^*^* |

Note: * means the shared location for *T. alexandrinum* and *T. resupinatum*, ^res^ means locations particular for *T. resupinatum*, the numbers of “Number” mean number of repeats in *T. alexandrinum* and *T. resupinatum*, respectively.
