# Supplementary material for: The Complete Chloroplast Genome of Two Important Annual Clover Species, Trifolium alexandrinum and T. resupinatum: Genome Structure, Comparative Analyses and Phylogenetic Relationships with Relatives in Leguminosae
Source: Plants (Basel). 2020 Apr 9;9(4):478. doi: 10.3390/plants9040478 (PMC7238141; doi:10.3390/plants9040478)
Supplement: Supplementary file 1 [file plants-09-00478-s001.zip › Table S3.docx]

Table S3 The relative synonymous codon usage (RSCU) analyzed using CodonW.

| AminoAcid | Symbol | Codon | No. | RSCU | AminoAcid | Symbol | Codon | No. | RSCU |
| --- | --- | --- | --- | --- | --- | --- | --- | --- | --- |
| * | Ter | UAA | 40 | 1.6215 | M | Met | AUU | 2 | 0.0129 |
| * | Ter | UGA | 20 | 0.8109 | M | Met | GUG | 2 | 0.0129 |
| * | Ter | UAG | 14 | 0.5676 | N | Asn | AAU | 786 | 1.5396 |
| A | Ala | GCU | 528 | 1.7836 | N | Asn | AAC | 235 | 0.4604 |
| A | Ala | GCA | 343 | 1.1588 | P | Pro | CCU | 345 | 1.5612 |
| A | Ala | GCC | 181 | 0.6116 | P | Pro | CCA | 250 | 1.1312 |
| A | Ala | GCG | 132 | 0.446 | P | Pro | CCC | 171 | 0.7736 |
| C | Cys | UGU | 175 | 1.5022 | P | Pro | CCG | 118 | 0.534 |
| C | Cys | UGC | 58 | 0.4978 | Q | Gln | CAA | 598 | 1.5946 |
| D | Asp | GAU | 664 | 1.5828 | Q | Gln | CAG | 152 | 0.4054 |
| D | Asp | GAC | 175 | 0.4172 | R | Arg | AGA | 375 | 1.8354 |
| E | Glu | GAA | 861 | 1.5106 | R | Arg | CGU | 285 | 1.395 |
| E | Glu | GAG | 279 | 0.4894 | R | Arg | CGA | 265 | 1.2972 |
| F | Phe | UUU | 899 | 1.4014 | R | Arg | AGG | 135 | 0.6606 |
| F | Phe | UUC | 384 | 0.5986 | R | Arg | CGC | 86 | 0.4206 |
| G | Gly | GGA | 588 | 1.6256 | R | Arg | CGG | 80 | 0.3918 |
| G | Gly | GGU | 504 | 1.3932 | S | Ser | UCU | 467 | 1.794 |
| G | Gly | GGG | 224 | 0.6192 | S | Ser | AGU | 317 | 1.2174 |
| G | Gly | GGC | 131 | 0.362 | S | Ser | UCA | 304 | 1.1676 |
| H | His | CAU | 364 | 1.5358 | S | Ser | UCC | 230 | 0.8832 |
| H | His | CAC | 110 | 0.4642 | S | Ser | UCG | 149 | 0.5724 |
| I | Ile | AUU | 939 | 1.5072 | S | Ser | AGC | 95 | 0.3648 |
| I | Ile | AUA | 606 | 0.9726 | T | Thr | ACU | 451 | 1.6384 |
| I | Ile | AUC | 324 | 0.5202 | T | Thr | ACA | 338 | 1.228 |
| K | Lys | AAA | 925 | 1.5664 | T | Thr | ACC | 190 | 0.6904 |
| K | Lys | AAG | 256 | 0.4336 | T | Thr | ACG | 122 | 0.4432 |
| L | Leu | UUA | 769 | 2.0166 | V | Val | GUU | 456 | 1.524 |
| L | Leu | CUU | 469 | 1.23 | V | Val | GUA | 448 | 1.4972 |
| L | Leu | UUG | 468 | 1.227 | V | Val | GUG | 153 | 0.5112 |
| L | Leu | CUA | 300 | 0.7866 | V | Val | GUC | 140 | 0.468 |
| L | Leu | CUG | 143 | 0.375 | W | Trp | UGG | 376 | 1 |
| L | Leu | CUC | 139 | 0.3648 | Y | Tyr | UAU | 646 | 1.6438 |
| M | Met | AUG | 465 | 2.9745 | Y | Tyr | UAC | 140 | 0.3562 |
