# Supplementary figures and images for: The Complete Chloroplast Genome of Two Important Annual Clover Species, Trifolium alexandrinum and T. resupinatum: Genome Structure, Comparative Analyses and Phylogenetic Relationships with Relatives in Leguminosae
Source: Plants (Basel). 2020 Apr 9;9(4):478. doi: 10.3390/plants9040478 (PMC7238141; doi:10.3390/plants9040478)

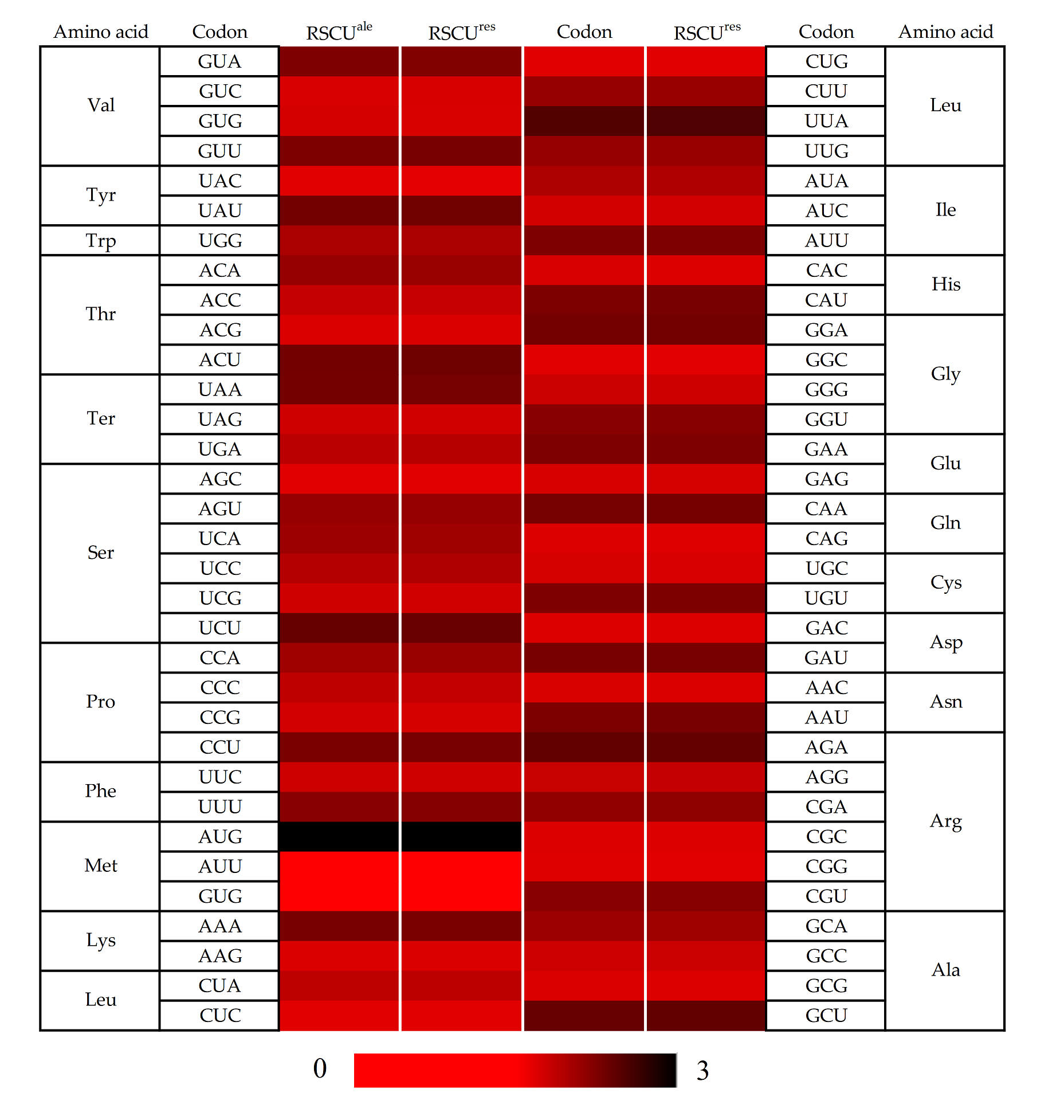

Supplement: Supplementary file 1 [file plants-09-00478-s001.zip › Fig S1.tif]

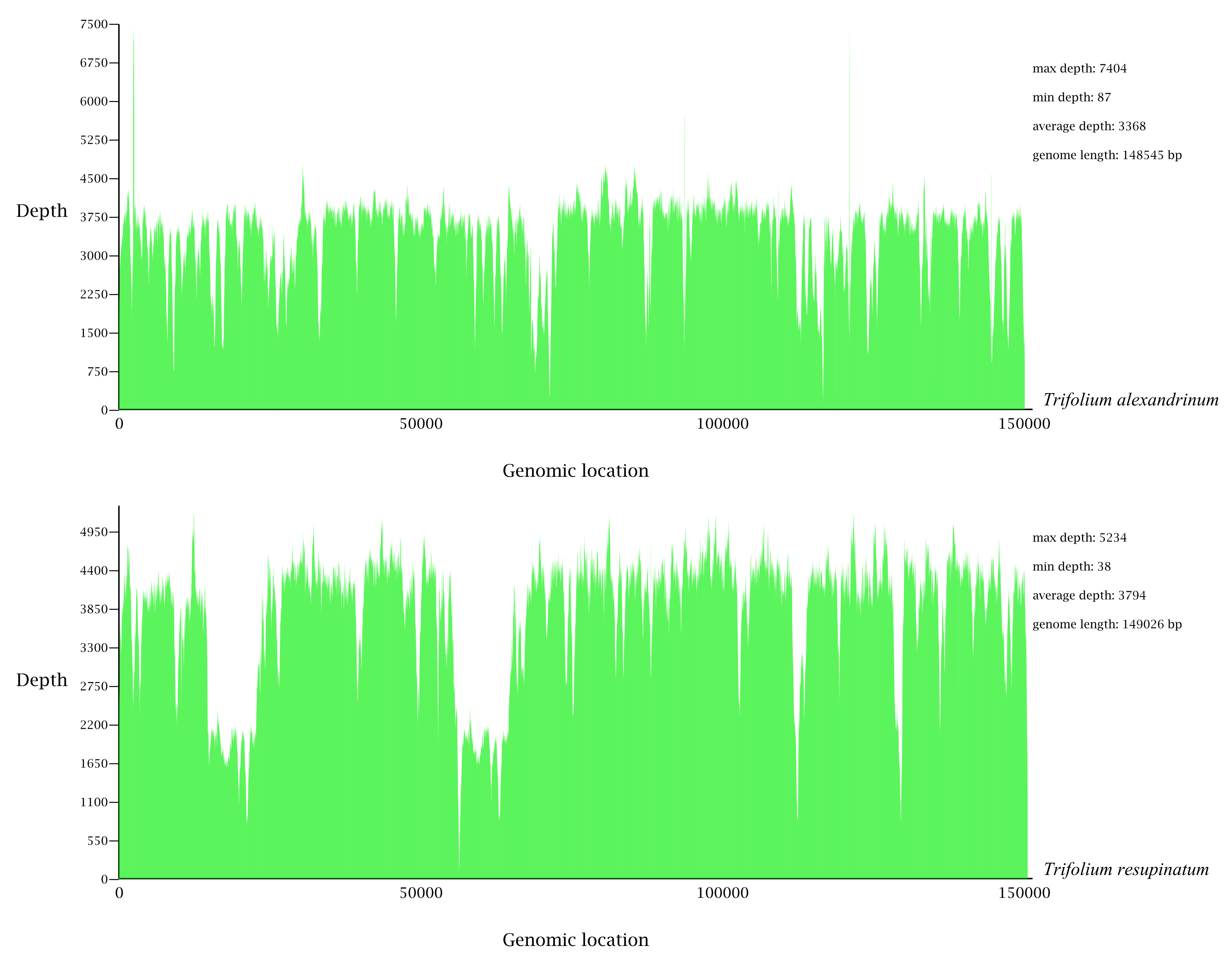

Supplement: Supplementary file 1 [file plants-09-00478-s001.zip › Fig S2.tif]
